# Determining salivary and serum levels of iron, zinc and vitamin B_12_ in patients with geographic tongue

**DOI:** 10.15171/joddd.2019.034

**Published:** 2019-10-07

**Authors:** Mina Khayamzadeh, Shamsoulmolouk Najafi, Parastoo Sadrolodabaei, Faranak Vakili, Mohammad Javad Kharrazi Fard

**Affiliations:** ^1^Department of Oral Medicine, School of Dentistry, Tehran University of Medical Sciences, International Campus, Tehran, Iran; ^2^Department of Oral Medicine, School of Dentistry, Tehran University of Medical Sciences, Tehran, Iran; ^3^Dentist, Private Practice, Tehran; ^4^Dental Student, School of Dentistry, Tehran University of Medical Sciences, International Campus, Tehran, Iran; ^5^Statistical Advisor, Dental Research Center, Tehran University of Medical Sciences, Tehran, Iran

**Keywords:** Blood, geographic tongue, iron, saliva, vitamin B_12_, zinc

## Abstract

***Background.*** Benign migratory glossitis or geographic tongue, whose cause still remains a mystery, emerges as annular lesions on the dorsal surface of the tongue. Several reasons have been reported for this condition, including vitamin deficiencies, digestive disorders, emotional stress and nutritional deficiencies. In order to introduce an efficient treatment for the geographic tongue, the present study investigated the levels of iron, zinc and vitamin B_12_ in the blood and saliva.

***Methods.*** The present study evaluated 40 subjects. The oral disease specialists examined the patients in the Dental School, Tehran University International Campus and Mashhad Dental School. Accordingly, inclusion and exclusion criteria were used to enroll the patients. The blood and salivary samples were collected from the case and control groups. Finally, independent t-test was used to analyze the data.

***Results.*** Overall, 20 subjects suffered from geographic tongue. They consisted of 8 females and 12 males, with a mean age of 33.5±4.8 and age range of 19‒49 years. Moreover, the mean age and age range of the healthy subjects were 29.40±7.5 and 24‒25 years, respectively. It should be pointed out that the subjects were equally divided into 10 males and 10 females. There was no difference between other variables in the blood and saliva.

***Conclusion.*** The results showed that patients with geographic tongue had lower levels of salivary zinc, compared to the control group. Although iron and vitamin B_12_ affect the lingual papillae, their serum and salivary levels did not change.

## Introduction

Geographic tongue is a common benign inflammatory condition, which occurs as annular lesions and is manifested as the loss of filiform papillae. These areas eventually become red and atrophy, surrounded by a yellowish-white prominent margin. The original location of geographic tongue is the dorsal tongue, affecting its sides in some cases.^1^

This lesion is often asymptomatic^2^ and therefore, patients are usually unaware of the lesion; however, sometimes it is sensitive to sour, hot and spicy foods and causes discomfort.^3^ The lesion requires treatment because of some problems such as pain, burning sensation, loss of taste, and fear of the patient about possible malignancy or precancerous lesion, as well as cosmetic concerns.^4,5^

In most studies, the prevalence of geographic tongue was reported between 1 and 5.2%,^6^ with a similar incidence in men and women.^7,8^ However, some studies have reported an occurrence ratio of 4:5 in women compared with men. The lesion may be found at any age.^9^

It is not yet clearly defined what causes geographic tongue. Several factors have been attributed for this condition, including nutritional deficiencies, hormonal disorders, use of oral contraceptives, juvenile diabetes mellitus, psoriasis, allergic conditions, fissured tongue, lichen planus, Down syndrome, mental factors, lithium consumption and a family history.^10^

Zinc deficiency is another factor associated with the lesion.^11^ Studies have mentioned the role of zinc in wound healing and maintaining a healthy epithelium, as well as reconstruction of filiform papillae.^12^ It was also reported that zinc deficiency can cause alterations in the oral epithelium and salivary composition, and atrophy of the tongue.^13^ Iron and vitamin B_12_ deficiency also appears to affect filiform papillae of the tongue. Geographic tongue is a lesion that affects the lingual papillae. It has been shown that zinc sulfate can positively contribute to the treatment of geographic tongue.^12^

Since no research has been carried out into iron, zinc and vitamin B_12_ levels in patients with geographic tongue, the present study aimed to evaluate these compounds in the blood and saliva of patients and compare them with a control group. The results of this study might provide an effective treatment for these patients and be a positive step toward protecting patient health and reducing the complications of untreated disease.

## Methods

In this case‒control study, the oral disease specialists examined 20 patients with geographic tongue at Tehran Dental School, International Campus and Mashhad Dental School, and 20 healthy subjects in terms of geographic tongue were enrolled in the study from September 2013 to February 2014, according to the inclusion and exclusion criteria. According to the criteria, the control group was selected from patients referring to the above-mentioned centers. The healthy group was investigated after enrolling each case. A pilot study was carried out on the case and control groups. For this purpose, two independent-sample tests were performed (α=0.05, β=0.2).

Geographical tongue, either symptomatic or asymptomatic, was approved in patients of the case group by oral disease specialists. The patients had not taken iron and vitamins during the last 8 weeks. However, pregnant women and people with systemic diseases, including severe disorders of the kidneys, psoriasis, Reiter’s syndrome, dermatitis, insulin-dependent diabetes and bronchitis, were excluded. The subjects in the control group did not have geographic tongue or the aforementioned systemic diseases and had not taken any medications. After filling out a consent form in both the case and control groups, 2 mL of venous blood were collected by 2-mL Supa disposable sterile syringes (with a needle gauge of 27) and poured into simple test tubes. The tubes were labeled with the subjects name, age and gender, and sent to the laboratory within 1 hour, where the blood was coagulated and the clot was separated from the serum by centrifugation (Biotech) at a speed of 7000 rpm for 25 minutes. The resultant serum was transferred into 5-mL transparent plastic tubes and stored at -20°C. The salivary samples were collected from 8:00 to 11:00 am through unstimulated spitting method (discharge of saliva every minute for 5 minutes) from all the subjects who had to refrain from eating and drinking and be relaxed for at least 2 hours prior to sampling. The samples were then poured into graded sterile plastic tubes which were immediately capped and sent to the laboratory along with the blood samples. The collected saliva was centrifuged for 15 minutes at 4500 rpm to separate insoluble materials and sputum and to obtain a clear fluid which was stored -20°C along with the serum samples until examination. The serum and salivary iron and zinc were measured through colorimetry using Pars Azmoon test kit (Iran) and Biorex kit (England, serial number BXC062A), respectively. Vitamin B_12_ was measured through chemiluminescene micro-particle immunoassay using AIA-PACK kit and TOSOH apparatus (Japan). The results were compared with normal ranges of the manufacturer. Independent t-test was used to compare the levels of zinc, vitamin B_12_ and iron between the healthy subjects and patients. Pearson’s correlation analysis was applied to determine the correlation between saliva and blood for each variable. All the statistical operations were performed using SPSS 20, considering a=0.05 and statistical significance of P<0.05.

## Results

Overall, 20 subjects suffered from geographic tongue. They consisted of 8 females and 12 males, with a mean age of 33.5±4.8 and an age range of 19‒49 years. Moreover, the mean age and age range of the healthy subjects were 29.40±7.5 and 24‒25 years, respectively. It should be pointed out that the subjects were equally divided into 10 males and 10 females (Table 1).

**Table 1:** The distribution of age and gender of patients and controls

**Group**	**Age**	**Mean age**	**SD**	**Female**	**Male**
**Case**	19‒49	33.5	4.8	8	12
**Control**	24‒45	29.4	7.5	10	10

The results showed no statistically significant difference in the serum levels of zinc between the case (97.35±13.51) and control (106.70±22.188) groups (P=0.11). The serum levels of iron in the case and control groups were 82.75±22.971 and 92.95±36.176, respectively, with no statistically significant difference (P=0.29). The serum level of vitamin B_12_ was 227.40±60.899 in the case group, with 232.30±85.483 in the control group, with no statistically significant difference (P=0.83). It was found that the levels of salivary zinc (0.136±0.070) were lower in the case group compared to the control group (0.233±0.113), which was significant (P=0.02). The salivary iron level was (0.157±0.081) in the case group, with (0.177±0.096) in the control group, exhibiting no statistically significant difference (P=0.27). The salivary level of vitamin B_12_ was (0.031±0.021) in the case group, with (0.038±0.016) in the control group, with no statistically significant difference (P=0.25).

Moreover, the results showed that 3 patients had lower levels of serum zinc, which was not normal (15%), while it was zero in the control group; 3 patients (15%) and 4 healthy subjects (20%) had iron deficiency; 11 patients (55%) and 10 healthy subjects (50%) had vitamin B_12_ deficiency; 15 patients (75%) and 6 healthy subjects (30%) had zinc deficiency; 7 patients (35%) and 8 healthy subjects (40%) had salivary zinc deficiency; and 5 patients (25%) and 2 healthy subjects (10%) had salivary vitamin B_12_ deficiency (Table 2).

**Table 2:** Comparison of the mean of the studied parameters in patients and controls

**Index**	**Group**	**Number**	**Mean ± SD**	***p*** **-value**	**Normal range**
**Serum iron (mg/dL )**	Case	20	82.75 ± 22.971	0.29	60‒160
	Control	20	92.95 ± 36.176		
**Serum zinc (m g/dL )**	Case	20	97.35 ± 13.51	0.11	70‒120
	Control	20	106.70 ± 22.188		
**Serum vitamin B**_12_ **(pg/mL )**	Case	20	227.40 ± 60.899	0.83	200‒900
	Control	20	232.30 ± 85.483		
**Saliva iron (ppm )**	Case	20	0.157 ± 0.081	0.27	0.14‒0.41
	Control	20	0.177 ± 0.096		
**Sali va zinc (ppm )**	Case	20	0.136 ± 0.070	0.02	0.017‒0.478
	Control	20	0.233 ± 0.113		
**Saliva vitamin B**_12_ **(ppm )**	Case	20	0.031 ± 0.021	0.25	0.02‒0.085
	Control	20	0.038 ± 0.016		

According to the Pearson’s correlation test, there was no significant correlation between the levels of serum and saliva iron (P=0.717), serum and saliva zinc (P=0.193), and serum and saliva vitamin B_12_ (P=0.158) (Table 3).

**Table 3:** Pearson’s correlation coefficient between the variables iron, zinc, and vitamin B_12_ in the serum and saliva

**Variables**	**Number**	**Correlation coefficient**	**P-value**
**Serum and saliva vitamin B**_12_	40	0.277	0.158
**Serum and saliva iron**	40	-0.059	0.717
**Serum and saliva zinc**	40	-0.210	0.193

## Discussion

**Figure 1:**
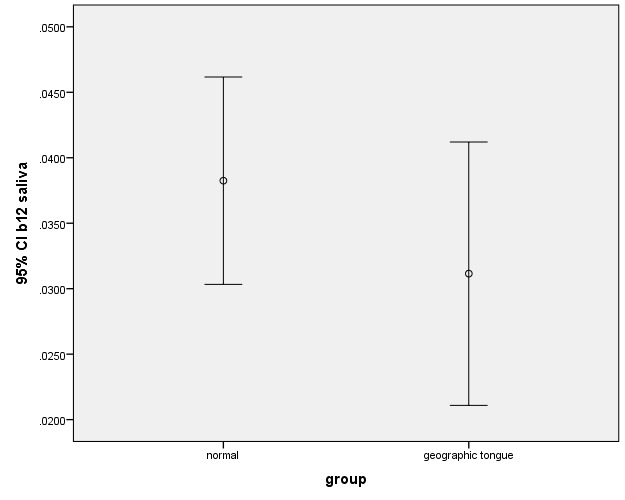
The error bars of the means and 95% confidence intervals of the means of salivary vitamin B_12_ levels in subjects with geographic tongue and healthy controls.

**Figure 2:**
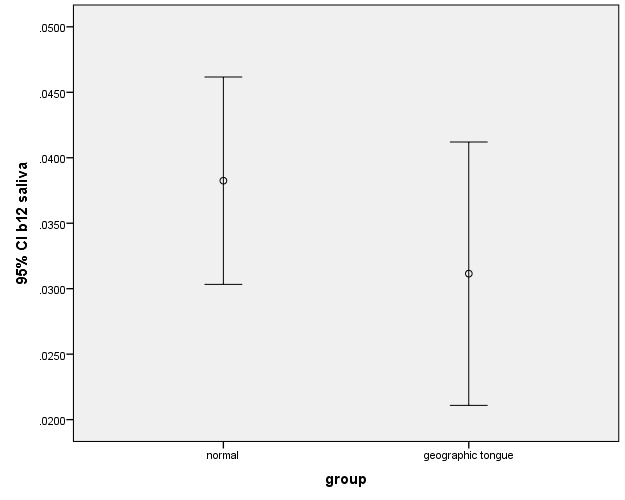
The error bars of means and 95% confidence intervals of the means of salivary iron levels in subjects with geographic tongue and healthy controls.

**Figure 3:**
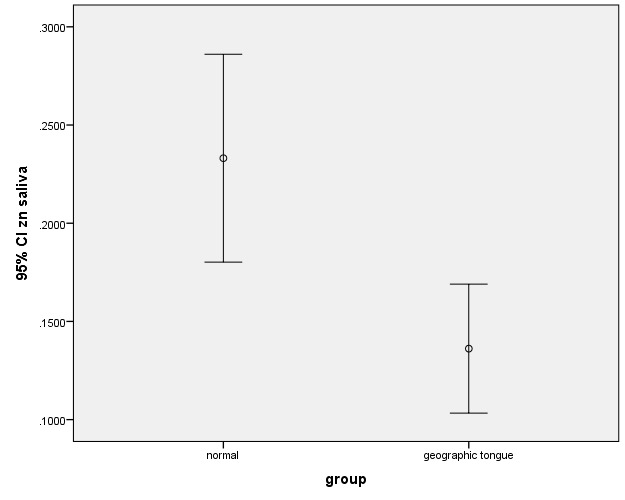
The error bars of means and 95% confidence intervals of the means of salivary zinc levels in subjects with geographic tongue and healthy controls.

In this study, there was no significant difference between the case and control groups in terms of the levels of zinc, iron and vitamin B_12_. There were also no significant differences between the salivary levels of vitamin B_12_ and iron (Figure 1, 2). However, the difference between the patients and controls in the salivary levels of zinc was statistically significant (Figure 3).

Different results have been reported about the prevalence of this lesion in terms of gender. Abdolsamadi et al^14^ reported a much higher prevalence in men compared with women (8.82% in males versus. 2.17% in females). Voros-Balog et al^15^ investigated the prevalence of geographic tongue. They found that it was more prevalent among males than females. However, Mumcu et al,^16^ Jainkittivong et al^17^ and Dos Santos^18^ showed that the geographic tongue was significantly more prevalent in girls than boys. Kulla-Mikkonen^18^ , Shulman and Carpenter^19^ and Kovac-Kovic et al^20^ reported that the geographic tongue was equally prevalent among males and females. Jainkittivong et al^17^ showed that the subjects at an age range of 20‒29 years suffered from this condition to the highest level (39.4%). In the present study, the levels of iron, zinc and vitamin B_12_ were studied in the blood and saliva in 20 patients, and according to the results, men were affected by geographic tongue at a higher rate than women. In order to introduce an efficient treatment for geographic tongue, the present study investigated the levels of iron, zinc and vitamin B_12_ in the blood. The patients suffering from geographic tongue consisted of 60% males and 40% females.

According to the clinical features of this lesion resulting from loss of filiform papillae, zinc deficiency can be related to the lesion given its role in the atrophy of lingual papillae or development of filiform papillae. The essential role of zinc in maintaining healthy epithelial tissues has been demonstrated in several clinical studies.^14^

In a research carried out on rabbits fed by a minimum amount of dietary zinc, they lost their filiform papillae, suggesting the effect of zinc on the development of lingual papillae.^20^

Abdolsamadi et al^14^ found that the patients with geographic tongue had lower levels of serum zinc, compared to the control group; this result is different from the results of our study. In the mentioned research, serum zinc levels in 6 patients with geographic tongue, out of 29, and 4 subjects in the control group, were lower than normal. While in our study, of 20 patients with geographic tongue, 3 had zinc levels lower than normal with normal levels of zinc in all the subjects in the control group. In addition, the results indicated that zinc deficiency can contribute to the filiform papillae atrophy, and it is probably associated with the development of filiform papillae.

In the present study, there was a statistically significant difference in the salivary zinc levels between the patients with geographic tongue and the controls. Abbas Al-Taee et al^21^ compared the levels of salivary zinc in patients with geographic tongue and the control group. They did not notice any significant difference.

No study has determined a specific and definite cause for the geographic tongue so far; however, various reasons have been mentioned, including emotional stress, nutritional deficiencies, allergies, heredity and genetics, immune deficiency, and systemic diseases such as diabetes.^12^

By evaluating the number of Langerhans cells and HLA-DP, -DQ and –DR expression in the epithelium of geographic tongue cases, Darling et al^22^ investigated whether geographic tongue is an antigen-driven condition. By quantifying the neurite area in the connective tissue of geographic tongue cases, peripheral nerve status was also evaluated for damage/injury association. According to the results, the number of Langerhans cells increased in the geographic tongue. Moreover, HLA expression was shown in the Langerhans cells, inflammatory cells, spinous layer and parabasal epithelial cells. According to the measurements, there was no significant difference between the patients and controls in terms of the total nerve tissue. Shulman and Carpenter investigated the prevalence and risk factors related to geographic tongue in US adults. They found that white and black people suffered from geographic tongue more than Mexican-Americans. This was inversely associated with smoking.^19^

Accordingly, zinc deficiency cannot solely be regarded as the main cause of geographic tongue, while zinc deficiency in saliva can only be proposed as a predisposing factor in the development of the lesion.

The present study investigated the relationship between iron, zinc and vitamin B_12_ in the blood and saliva for the first time according to our literature search. Accordingly, no correlation was found between the mentioned variables in the saliva and blood; therefore, no significant difference was seen in the serum levels of zinc between the case and control groups; however, this difference was found in the saliva.

The presence of a significant difference in the salivary zinc levels between the patients and controls and the absence of this difference in the serum might reflect changes occurring in the salivary glands and the composition of saliva in patients with the geographic tongue; however, further investigations are necessary.

According to the results, investigations are necessary into the saliva of further samples. The administration of systemic or topical zinc, such as mouthwashes, can also be investigated as a treatment option for the geographic tongue. Due to the presence of this lesion in children, further surveys about its possible causes in children can also be performed.

## Conclusion

According to the results, vitamin B_12_ and iron deficiency can contribute to the filiform papillae atrophy, and it is probably associated with the development of filiform papillae. Although there were no differences in the serum zinc levels between the case and control groups, its levels were lower in the saliva of the case group, which confirms the impact of saliva zinc deficiency in the development of geographic tongue, particularly when considering the positive effect of zinc sulfate administration on improving the filiform papillae in patients with geographic tongue. However, other factors can certainly be effective in the development of this disease because zinc deficiency in the saliva has not been seen in all the patients.

## Competing Interests

The authors declare no conflict(s) of interest related to the publication of this work.

## Authors’ Contributions

MK designed the study, wrote the manuscript and revised the article. SN revised the manuscript. PS and FV wrote the manuscript and collected data. MJKF analyzed the data.

## Acknowledgments

This study could not be carried out without co-operations from Tehran Dental School, International Campus and Mashhad Dental School. We are also grateful to our study group for their willingness to participate in the study and for their support.

## Funding

This study was funded by the Dental Research Center, School of Dentistry, International Campus, Tehran University of Medical Sciences.

## Ethics Approval

This study has ethical committee approval provided by "Dental research center of Tehran University of Medical Science."
